# Comparison of Radial Extracorporeal Shock Wave Therapy in Plantar Fasciitis Treatment Using Two Different Frequencies

**DOI:** 10.7759/cureus.8284

**Published:** 2020-05-26

**Authors:** Selnur Narin, Bayram Unver, Nihat Demirhan Demirkıran, Mehmet Erduran

**Affiliations:** 1 Physiotherapy and Rehabilitation, Institute of Health, Dokuz Eylul University, Izmir, TUR; 2 School of Physical Therapy and Rehabilitation, Dokuz Eylul University, Izmir, TUR; 3 Orthopaedics, Kütahya Health Sciences University School of Medicine, Kütahya, TUR; 4 Orthopaedics and Traumatology, Dokuz Eylul University, Izmir, TUR

**Keywords:** heel pain, plantar fasciitis, shockwave, radial shockwave therapy

## Abstract

Objective

To compare results of two different frequencies and densities of radial extracorporeal shock wave therapy (rESWT) after 10 sessions.

Methods

A total of 41 patients with plantar fasciitis were included in this study. Patients were randomly divided into two groups. Both groups were administered 10 sessions of treatment consisting of 15 Hz frequency, 3.0 Bar density and 2000 impulses/ session for the 1^st^ group, and 10 Hz frequency, 2.0 Bar density and 2000 impulses/ session for the 2^nd^ group. Visual analog scale (VAS) and a modification of the clinical rating system of the American Orthopedic Foot and Ankle Society (AOFAS) were used for outcome measurement. The patients were assessed before treatment and followed up four weeks, and 12 weeks after end of treatment.

Results

Mean VAS scores were reduced after rESWT from 7.52 ± 2.34 (mean ± SEM) at baseline to 0.57 ± 0.68 at 12 weeks in the 1^st^ group and from 6.45 ± 2.04 at baseline to 0.40 ± 0.60 at 12 weeks in the 2^nd^ group. Similar changes were found for mean AOFAS scores from baseline after rESWT but were not observed significance between groups.

Conclusion

There is no significant different effect between the two treatment groups’ results.

## Introduction

Plantar fasciitis (PF), the most common cause of heel pain, accounts for approximately 11-15% of foot symptoms requiring professional care in the adult patient group [1-3]. Present conservative treatment options for plantar fasciitis include physical therapy, nonsteroidal anti-inflammatory drugs, corticosteroid injections, taping, orthotics, shoe, and activity modifications, night splinting, and casts [4]. Extracorporeal shock wave therapy (ESWT) has been proposed as a potential method of treating patients with chronic diseases without the need to prevent weight-bearing [5]. Several controlled trials of ESWT for chronic PF have been published demonstrating favorable results in the range of 50% to 70% of patients after a follow-up period of three months after treatment [3,6-9]. Besides, a recent study demonstrated the safety and efficacy of radial extracorporeal shock wave therapy (rESWT) for chronic PF [3].

To further evaluate the potential of rESWT to become a routine therapeutic modality in the treatment of chronic PF, we identified the following questions not addressed in the study by Gerdesmeyer et al. [3]. First, it is unknown whether treatment success can also be reached by two rESWT sessions one week apart, rather than by three rESWT sessions each two weeks apart as applied by Gerdesmeyer et al. [3]. There is still much debate over several issues surrounding shock wave therapy (SWT) that have not been adequately addressed by the literature: high versus low energy SWT, shock wave dosage and the number of sessions required for therapeutic effects [6].

ESWT is painful, may aggravate symptoms for a short period of time and may induce reversible local swelling and formation of hematoma [7]. rESWT make use of Newton’s third law (action and reaction) and are generated through the action of an air compressor. These waves are transmitted radially, with the greatest energy in the surface region of the skin and decrease gradually on deeper tissues. Its biological effects (cavitation, neovascularization, and analgesia) are similar to those of other wave generators, but the physical characteristics are different. Radial waves are used preferentially in cases of plantar fasciitis, lateral epicondylitis (tennis elbow), patellar tendinitis, trochanteric bursitis, calcified tendinitis of the shoulder, calcaneus tendinitis and, most recently, the trigger points in myofascial syndromes. Radial shockwaves are used in cases of soft-tissue diseases and in more superficial locations [8,10-12].

The objective of this study was to provide other elements in the search for an optimal protocol in the treatment of painful subcalcaneal spurs, as the need for future researches to ascertain the most beneficial protocol for patient care was emphasized in the literature. Therefore, we planned our study hypothesis to compare 10-day rESWT results in two different frequencies and densities. Our results may shed light to new methods in rESWT application.

## Materials and methods

Patients

This single-blind, randomized, controlled trial group design was conducted at our University Hospital and Physical Therapy School. A total of 41 patients with unilateral, chronic PF were enrolled in the present study between June 2015 and October 2016. Patients were diagnosed as chronic PF by orthopedic surgeons based on the patient’s history and physical findings including heel pain and local tenderness over the plantar-medial aspect of the calcaneal tuberosity near the plantar fascia insertion. Radiographs showed the presence of a heel spur in 81% of the patients. Patients were referred to the principal investigator in the School of Physical Therapy and considered for participation in the present study according to the inclusion and exclusion criteria summarized in Table 1.

**Table 1:** Inclusion and exclusion criteria of patients with chronic plantar fasciitis enrolled in the present study

Inclusion criteria	Exclusion criteria
Adults over the age of 18 years	Bilateral plantar fasciitis
Diagnosis of painful heel syndrome by clinical examination, with the following positive clinical signs:	Dysfunction of foot or ankle (for example, instability)
1. Pain in the morning or after sitting a long time	Arthrosis or arthritis of the foot
2. Local pain where the fascia attaches to the heel	Infections or tumors of the lower extremity
3. Increasing pain with extended walking or standing for more than 15 minutes	Neurological abnormalities, nerve entrapment (for example, tarsal tunnel syndrome)
History of 6 months of unsuccessful conservative treatment	Vascular abnormality (for example, severe varicosities, chronic ischemia)
Therapy-free period of at least 4 weeks before referral	Operative treatment of the heel spur
Signed informed consent	Hemorrhagic disorders and anticoagulant therapy
	Pregnancy
	Diabetes

The average number of patients with a heel spur that our University Hospital’s Orthopedics and Traumatology doctor examines/can examine per month was gathered to be used as patient population [2,6]. The minimum number of sample size was determined using sample size calculation suitable for randomized control research with StatCalc (EpiInfo, Version 6) program. The number of patients was determined by power analysis. Population was determined as 10 out of 50 patients on average, with 95% confidence interval, 10% frequency, and 5% error margin calculated with minimum sample size 40. Using 40-45 patients was planned based on literature research. ‘Completed randomization’ method was used in randomization. No patient dropped out from the study after randomization. Ethical approval was obtained from the University Hospital Ethical Committee before starting the study. Written informed consent was obtained from all participants.

Treatment

**Figure 1:**
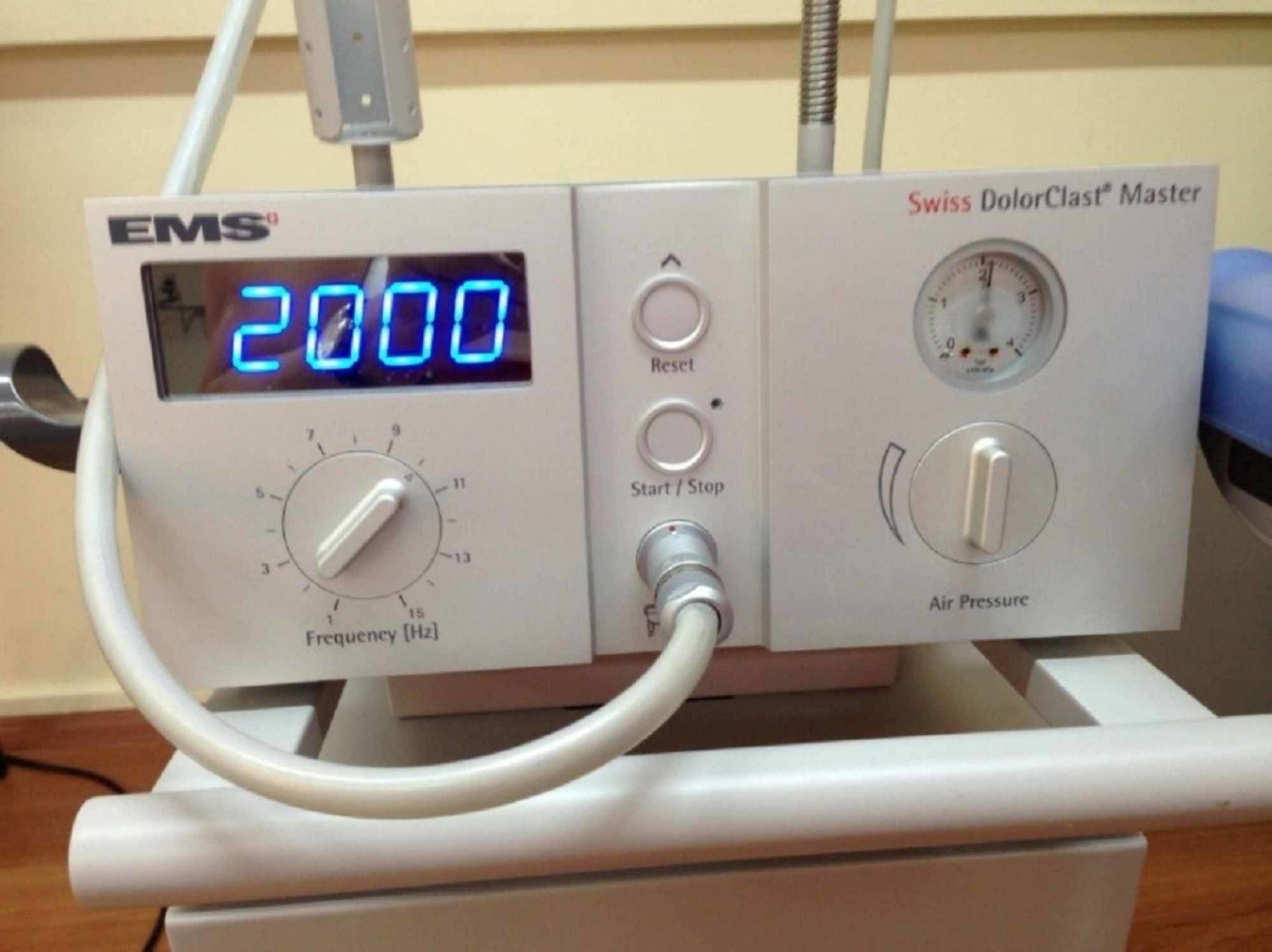
EMS Electro Medical Systems device that was used in the treatment

rESWT was performed by the principal investigator with the EMS Swiss DolorClast® (EMS Electro Medical Systems Corporation, Dallas, TX, USA) shown in Figure 1.

In group 1, each patient received 10 sessions of rESWT, with 2,000 impulses per session, air pressure of the device set at 3.0 bar; impulses applied with the 15-mm applicator at a frequency of 15 Hz. In group 2, patients received 10 sessions of rESWT, with 2,000 impulses per session, air pressure of the device set at 2.0 bar; impulses applied with the 15-mm applicator at a frequency of 10 Hz. The patients were not aware whether they enrolled to rESWT group I or group II treatment. Local anesthesia was not applied. No other (conservative) treatments were allowed during the study [7].

Outcome evaluation

The evaluations were made before and immediately after the treatment, four and 12 weeks after the end of the treatment. The evaluations were conducted by the same therapist. Pain intensity was measured using visual analog scale (VAS) score (We used a 10-cm visual analog pain scale with 0 being no pain and 10 being maximal pain), the function of the foot was measured by American Orthopedic Foot and Ankle Society (AOFAS) score (pain and range of motion domains), a validated rating scale which incorporates assessment of function (50%), pain (40%), and alignment (10%) [12]. The AOFAS clinical rating system consists of subjective and objective variables, the objective clinical component is scored by the physiotherapist and the subjective questions are answered by the patient. A complete description of the AOFAS clinical rating scales and scoring methodology has been previously reported and indicative of the clinical rating scales' ability to discriminate and predict the quality of life related to foot and ankle conditions [8,9]. And the validity and reliability study was conducted for AOFAS [13].

Also, heel sensitivity (with palpation), ankle range of motion (dorsiflexion and plantar flexion with a goniometer), strength tests (dorsiflexion, plantar flexion with manual muscle test) were evaluated before and after treatment.

Statistical methods

For the patients, mean of the VAS and the AOFAS scores were calculated for each time point (i.e., at baseline as well as four weeks, 12 weeks after rESWT). Comparisons between groups treatment were performed using Mann-Whitney U test, followed by Bonferroni post-tests to compare replicate means by the investigated time points. In all analyses, an effect was considered statistically significant if its associated p-value was smaller than 0.05. Calculations were performed using SPSS, version 15.0.0 for Windows (SPSS, Chicago, IL, USA).

## Results

All patients enrolled in the present study finished the corresponding treatment. Twenty-one feet treated in group I and 20 feet in group II. No patients were needed to receive any other type of treatment during the follow-up. The demographic characteristics of patients are shown in Table 2. There were no significant differences between patients regarding to age and BMI (p > 0.05). Statistically significant difference was not observed between the gender distribution, background stories and family histories of group II (p > 0.05). Also, there was no difference in intergroup right / left extremity distribution (p: 0.44).

**Table 2:** Characteristics of groups I and II with confirmed diagnosis of the plantar fasciitis

		Male	%	Female	%	Age (yrs) (Mean ± SD)	BMI (kg/m^2^) (Mean ± SD)
Group I		6	28.6	15	71.4	49.05 ± 8.86	28.51 ± 3.86
Group II		16	55	4	45	50.50 ± 13.87	27.51 ± 2.80

With the numbers available, the group I patients (n = 21) treated with rESWT were not significantly different from the group II patients (n = 20) with respect to the sex distribution, mean age, mean body weight, affected side and types of job (Table 2).

**Figure 2:**
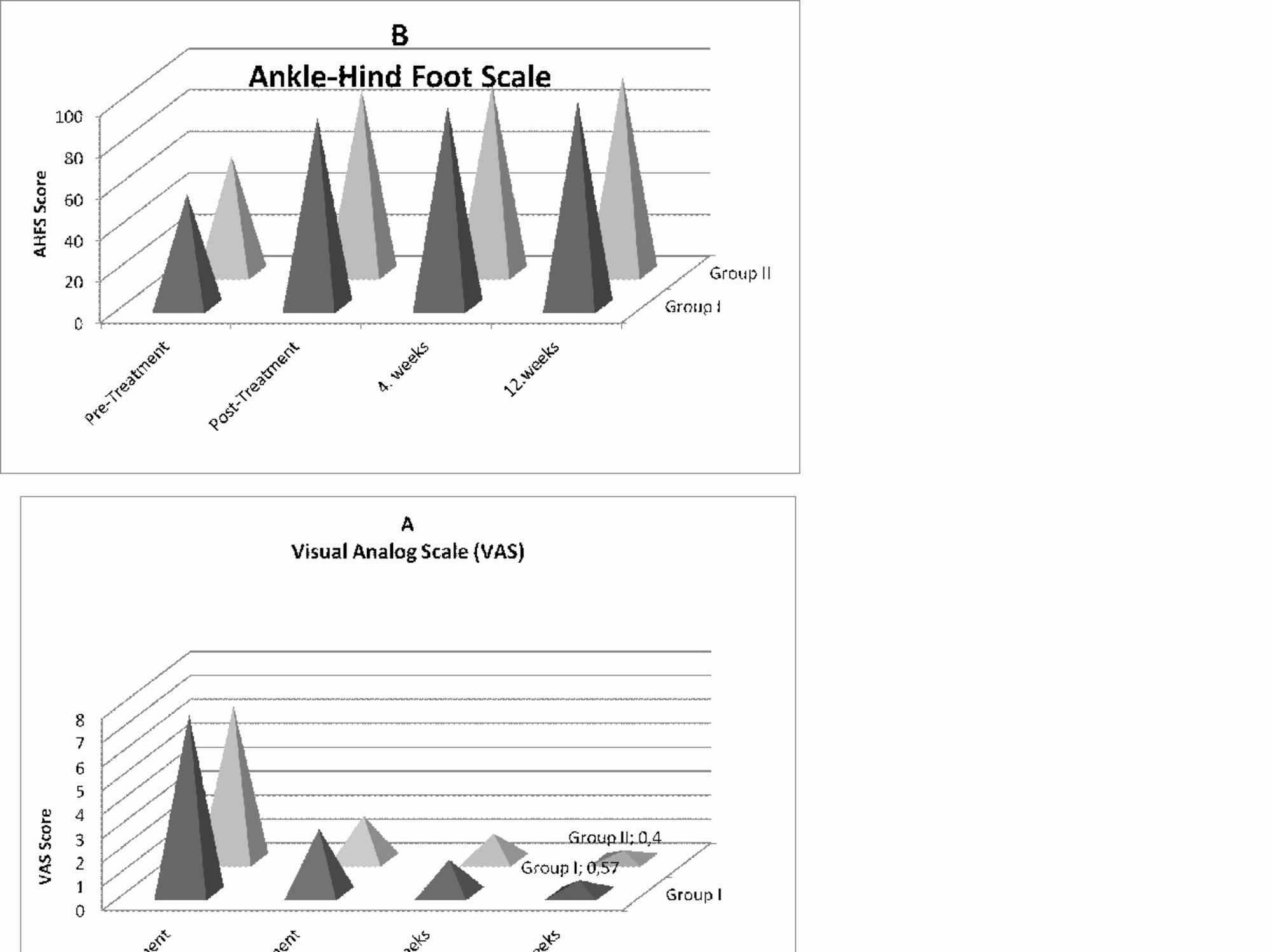
Mean and standard error of the mean of visual analog scale (VAS) scores (A) and ankle-hindfoot scale (AHFS) scores (B) of patients with chronic plantar fasciitis after treatment with radial extracorporeal shock wave therapy (rESWT; n = 21; group I) and group II (n = 20; open bars) at pre-treatment, post-treatment, four weeks and 12 weeks after the first rESWT p > 0.001.

When comparing the pre-treatment mean values of pain and foot function for the two groups, the results did not reveal any differences (VAS, P = 0.117 and 0.340, respectively). VAS and AOFAS results of both groups improved significantly between pre- and post-treatment, 4th week and 12th week. However, the comparison between post-intervention scores of pain and foot function also showed a non-significant difference between the two treatment groups (Table 3). The variation differences occurring in VAS and AOFAS in both groups were similar. Statistically significant difference was not observed (Table 3, Figure 2).

**Table 3:** Bonferroni post-tests for pain (VAS) and function of the foot (AOFAS) studied groups

		Group I (n = 21)	Group II (n = 20)	
		Mean ± SD	Mean ± SD	p-value
Pain (VAS)	Pre-treatment	7.52 ± 2.34	6.45 ± 2.04	0.12
	Post-treatment	2.71 ± 1.74	1.85 ± 1.66	0.14
	4 weeks	1.43 ± 1.21	1.10 ± 1.02	0.37
	12 weeks	0.57 ± 0.68	0.40 ± 0.60	0.39
AOFAS	Pre-treatment	54.57 ± 13.07	56.65 ± 13.39	0.34
	Post-treatment	91.71 ± 7.16	88.50 ± 8.15	0.23
	4 weeks	97.05 ± 3.50	90.80 ± 5.63	0.01
	12 weeks	99.24 ± 1.48	95.30 ± 5.35	0.04

When heel sensibility, ankle range of motion (dorsiflexion and plantar flexion) and muscle strength tests were evaluated pre- and post-treatment within the group and between the groups, statistically significant difference was not observed (p > 0.005).

## Discussion

The present study demonstrated significant improvement in pain level and functional measurement, after radial extracorporeal shock wave therapy (rESWT) at follow-up compared to baseline in both groups after 10 sessions.

Several studies have been conducted to investigate the effects of extracorporeal shock wave therapy on treated tissue, such as favorable prognostic factors in treatment outcome, comparison between extracorporeal shock wave therapy and corticosteroid injection [12,14-16]. Also, treatment protocols differ according to energy-flux density, the number of sessions and type of devices. Given the variety of protocols and equipment, it is currently impossible to establish the superiority of one over the other [17].

It is difficult to compare studies, which use different patient populations, design types of devices and treatment protocols. It is unclear if the negative results of other studies are due to insufficient energy levels, possible overtreatment, which can produce a lack of/or negative biologic effect, or inclusion of patients who might not benefit from rESWT.

A lot of studies find good results after the application of focused ESWT in patients with plantar fasciitis with success rate ranging from 34% to 88%, which is consistent with our findings. There are also different results about the effectiveness of ESWT in the treatment of plantar fasciitis in the literature, it is considered to be a safe and effective method in the treatment of chronic plantar fasciitis and it is recommended for patients who have heel pain for more than three months and who do not respond to conservative treatment. Ogden observed in his study that ESWT application was effective in plantar fasciitis, and suggested that it should be applied before any surgical treatment and even might be preferred to the cortisone injection [18]. Also, Gerdesmeyer et al. concluded that ESWT was effective on pain, function and quality of life when compared with placebo in patients with persistent plantar fasciitis [3].

Rompe et al. compared two different ESWT protocols applied to 112 patients in a randomized controlled clinical study they carried out [19]. Within two weeks, 1000 shot 3 applications to 1st Group and 10 shot 3 applications to 2nd group were performed. In VAS score after six weeks, while a decrease was observed from 77 to 19 in the 1st group, a significant decrease was not observed in the 2nd group. Rucker, in her study, applied three sessions of ESWT treatment in total by one-week intervals consisting of 15 Hz frequency, 20 Bar density, and 2000 impulse/session [20]. After the application, a significant decrease was observed in VAS from 75 to 49 in the first week, to 38 in the second week and to 23 in the third week. The decrease between the third week and 3rd month decreased from 23 to 20, and this was not observed statistically significant. With the obtained result, no difference was observed between groups despite the low energy repetitive application of ESWT and obtaining successful results in the short and medium-term. In our study, in group I each patient received 10 sessions of rESWT, with 2,000 impulses per session, air pressure of the device set at 3.0 bar; impulses applied with the 15-mm applicator at a frequency of 15 Hz (Figure 2). In group II, each patient received 10 sessions of rESWT, with 2,000 impulses per session, air pressure of the device set at 2.0 bar; impulses applied with the 15-mm applicator at a frequency of 10 Hz. Although our VAS values decreased significantly pre-post treatment and after 12th week (group 1; 7.5 / 2.7/ 0.57; group 2: 6.45/ 1.85/ 0.40) in both groups, the difference between groups was not statistically significant. Our results, in compliance with the literature, showed that rESWT was effective on heel pain and symptoms even in the third month after treatment, more decrease in VAS values was observed.

In the studies, there are different energy and impulse applications with ESWT [3,21]. In our study, the reason of applying two doses and frequency different from the studies carried out in the literature was to examine these effects and to determine the optimal protocol. However, there are also studies reporting that ESWT is ineffective in the treatment of chronic plantar fasciitis, and therefore, has no effect on pain, function and the quality of life [22-24].

It was stated that different results in the literature might result from the differences in study methodology such as possible patient selection criteria, the use of different devices, different energy levels and the total energy and outcome measures [21]. The indications for application in chronic disorders with a history of more than six months should be observed [8]. Also in our study, the fact that the cases we took had complaints for at least six months was the criterion.

In the present study, no complications resulting from the use of rESWT were observed. Plantar fasciitis is often bilateral, and in our study, no cases had this condition in both feet because it is in our exclusion criteria [7]. Women are affected more than men. Plantar fasciitis is associated with obesity and climacteric syndrome. Similarly, in the present study, women were more affected: in group I, 15 female patients (71.4%) vs. six male patients (28.6%); in group II, 16 female patients (80%) vs. four male patients (20%). In our study, 1/5 of the patients were overweight in both groups. It was similar in terms of weight distribution; however, there were no numbers of patients at the rate of supporting the relationship of the heel pain and overweight. Twenty-two patients (53%) in this study had not undergone any previous treatments.

Instead of Roles & Maudsley (RM) score, we used the American Orthopedic Foot and Ankle Society (AOFAS) rating system, which is more specific for foot disorders [25]. We could not make a direct comparison as different scorings were used in the studies. The results of our study are parallel to the literature. At the 12-week follow-up, both treatment protocols were effective for improving functional ability among the patients with heel pain. The improvement with group 2 was faster.

Different mechanisms of action of different energy density levels have been hypothesized for the reduction in pain symptoms after extracorporeal shock wave therapy. Experimental studies have shown that shock waves act selectively on nonmyelinated peripheral sensitive fibers, without interfering with motor nerve fiber activity. At high-energy levels, selective destruction of these fibers in the focal area of treatment could contribute to prolonged analgesia. In low-energy treatment, the mechanism underlying analgesia may be attributed to the local release of neuropeptides, leading to neurogenic inflammation and prevention of reinnervation by sensitive local nerve endings. Repeated application is thought to promote inflammation, thus preventing reinnervation [17,26-30].

## Conclusions

The results of our randomized controlled study revealed no differences between two groups receiving rESWT with two different frequencies for plantar fasciitis treatment. Although the lower frequency group leads to a faster improvement, this difference was not statistically significant. Both protocols were found effective for improving pain and functional scores at four- and 12-week follow-up periods.
